# Real-Time Detection of IoT Anomalies and Intrusion Data in Smart Cities Using Multi-Agent System

**DOI:** 10.3390/s24247886

**Published:** 2024-12-10

**Authors:** Maria Viorela Muntean

**Affiliations:** Department of Informatics, Mathematics and Electronics, 1 Decembrie 1918 University of Alba Iulia, 510009 Alba Iulia, Romania; mmuntean@uab.ro

**Keywords:** IoT data, cybersecurity, smart city, multi-agent system, learning model, decision rules, unbalanced dataset

## Abstract

Analyzing IoT data is an important challenge in the smart cities domain due to the complexity of network traffic generated by a large number of interconnected devices: smart cameras, light bulbs, motion sensors, voice assistants, and so on. To overcome this issue, a multi-agent system is proposed to deal with all machine learning steps, from preprocessing and labeling data to discovering the most suitable model for the analyzed dataset. This paper shows that dividing the work into different tasks, managed by specialized agents, and evaluating the discovered models by an Expert System Agent leads to better results in the learning process.

## 1. Introduction

Smart cities leverage advanced technologies, data analytics, and interconnected systems to develop efficient, sustainable, and livable environments. According to [1], the most important key components of a smart city are smart city architecture (perception layer, network layer, application layer), smart city applications (smart grids, smart lighting, smart parking, smart buildings, smart healthcare, public security, smart waste management, smart surveillance system, smart food distribution, smart water distribution, smart manufacturing system, smart transportation systems), and smart city challenges (sensor networks, communication challenges, governance challenges, security, big data).

By connecting devices and sensors, cities can gather and analyze data in real-time, optimizing the offered services. The Internet of Things (IoT) is used to enhance urban living, allowing remote management and access to the generated data. However, the increased number of IoT interconnected devices and sensors leads to an increased number of vulnerabilities, especially in terms of cybersecurity. An important challenge for the cities is to protect their critical infrastructure from potential cyber threats.

The potential threats can be detected by analyzing the generated IoT traffic data. Due to the large amounts of collected data, different approaches based on machine learning are proposed.

Ishaani Priyadarshini applies federated learning and split learning [2] and generates learning models for edge devices that are connected through IoT. The proposed approach minimizes privacy risks, minimizes latency, and reduces network bandwidth usage. The local and the global models were built using different machine learning techniques such as naïve Bayes, logistic regression, decision trees, random forests, extreme gradient boosting, artificial neural networks, convolutional neural networks, long short-term memory, and support vector machines.

Models such as convolutional neural networks, artificial neural networks, long short-term memory, and gated recurrent units were also proposed in [3]. The authors use a hybrid approach to accurately detect malicious actions based on attack patterns.

A novel approach based on convolutional neural networks is proposed in [4], where the developed model records an accuracy rate of 99% for all the considered attack categories. 

Machine learning and deep learning techniques proved to be efficient in intrusions and anomaly detection within an IoT network, according to Saida Hafsa Rafique et al., which summarizes the current literature [5]. Datasets and benchmarks for anomaly detection are proposed in [6].

A dual model that identifies flooding attacks is proposed in [7]. The first model performs predictions using regression algorithms, such as linear regression, support vector regression, and decision trees, while the second model introduces a degree of noise. The advantage of the proposed architecture is that it transforms the non-linear data into a more suitable format to identify unusual data points.

Burhan Ul Islam Khan et al. propose to enhance data security by integrating AI and blockchain technologies [8]. The proposed system architecture includes extracting sensory data, legitimacy evaluation, blockchain authentication and transformation, abnormality detection, and then building the AI model while also consulting cloud storage units. The proposed framework was validated with different datasets and demonstrated an improved detection accuracy of 8.1% compared to other AI models. 

Other approaches in the field of smart cities include forecasting methods [9], traffic congestion models [10], and a multi-box detector at urban intersections [11].

Multi-agent systems for IoT data include service provisioning [12] and data sampling and transmission reduction [13]. An agent-based perspective for learning IoT data is also described in [14,15].

Previous works proposed multi-agent systems for learning ontology-based documents (with six categories of agents: information updater agent, document uploader agent, parser agent, convertor agent, clustering agent with k-means partitioning method, and subset extractor agent) [16] or automating urban traffic management processes (where the following agents were modeled: traffic flow agent, road junction agent, and car parking agent that used KNN forecaster learning method, fault detection agent that incorporated decision trees classifier, and monitoring agent) [17].

In this research, I propose a multi-agent system that analyzes and real-time detects intrusions and anomalies in IoT traffic data in order to avoid unauthorized access to the interconnected devices. Leaning IoT data is time-consuming due to the large number of features, so preprocessing and clustering methods are mandatory in these applications.

The model optimizes the true negative rates of IoT traffic data using an Expert System Agent that identifies with high performance the instances with abnormal behavior. The learning process is modeled through intelligent agents, automating all machine learning steps, from data preprocessing and data clustering to data classification and meta-classification. The proposed system is suitable for real-time data monitoring, and it learns unbalanced datasets with high accuracy rates and in optimum time.

## 2. Multi-Agent System for Real-Time Learning of IoT Data: Meta-Classification Based on Decision System Model

**Figure 1:**
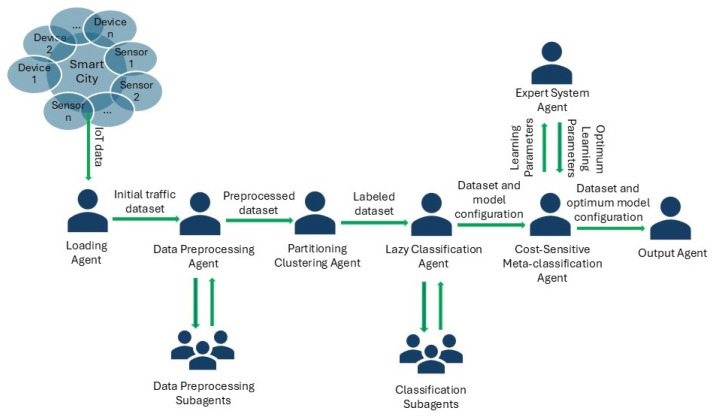
Multi-agent system architecture for IoT data.

The proposed system architecture is suitable for IoT traffic data and focuses on anomalies and intrusions detection in real-time. The system is composed of seven agents and six subagents with specific tasks (Figure 1). 

The proposed agents communicate with each other and change information in terms of datasets and optimum values for parameters of the learning methods. The learning methods were chosen after performing a series of experiments that will be described in the next section. The integrated methods proved to be the most suitable ones for the IoT traffic dataset considered as a case study. Agent behaviors are listed in Table 1.

**Figure 2:**
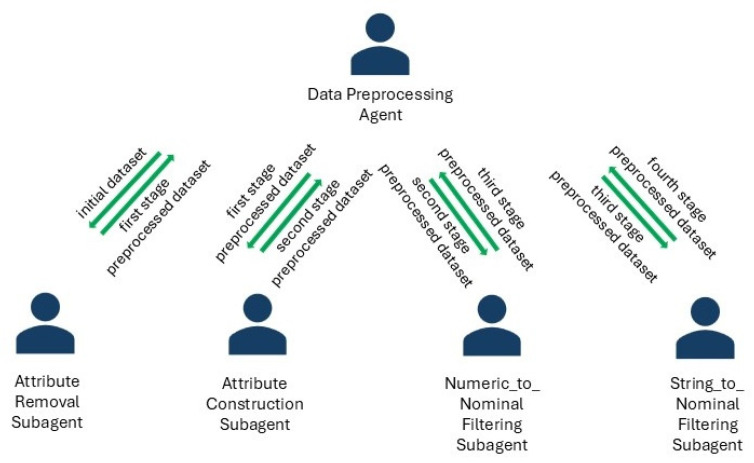
Data preprocessing system architecture for IoT data.

**Figure 3:**
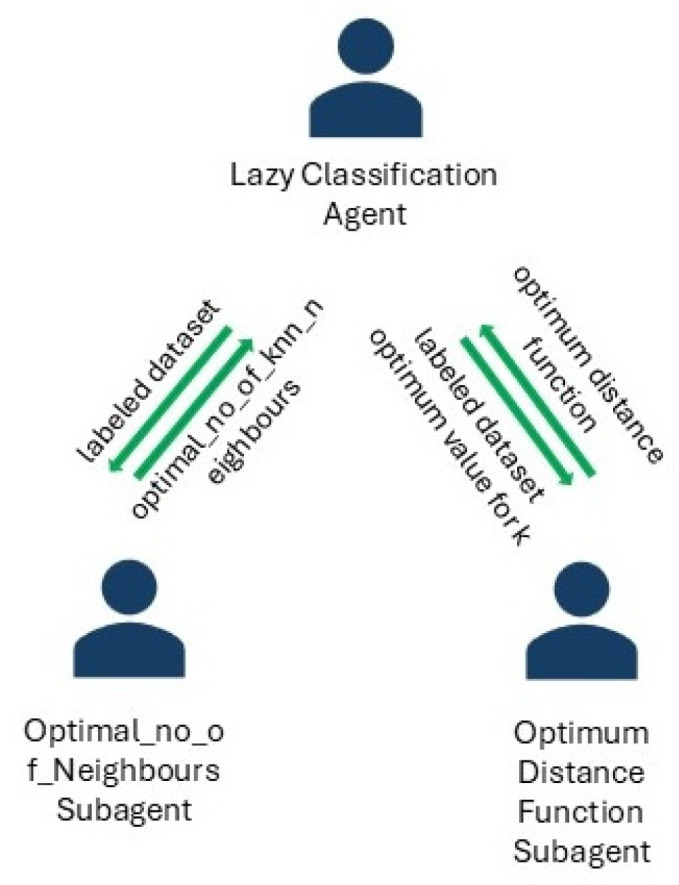
Data classification system architecture for IoT data.

The proposed system contains two subagent teams (Figure 2 and Figure 3) for improving preprocessing and learning processes by identifying the optimum dataset structure and the optimum configuration for the discovered models.

Subagents communicate, receive, and send data to their chief agent and also have specific behaviors that are presented in Table 2.

The results received at each proposed task, together with the dataset used and the proposed expert system, are described in the next section.

## 3. Experimental Results

**Figure 4:**
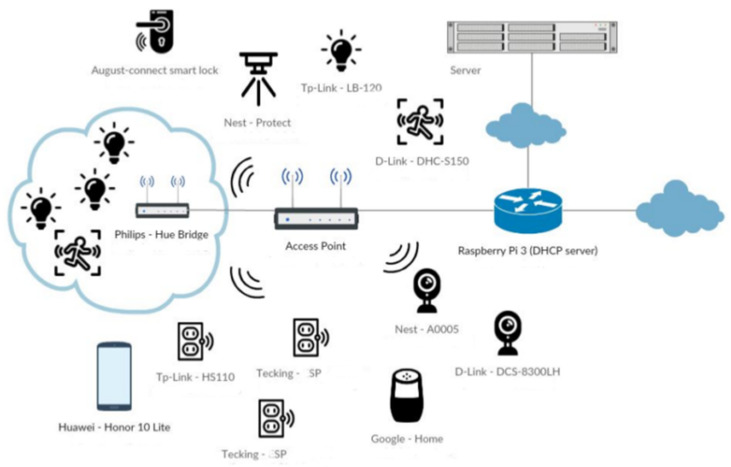
The architecture for IoT data generation and collection proposed in the VARIoT project [21].

The dataset used for this research was obtained from [18,19] and stores IoT network traffic data generated by interconnected devices and sensors. The dataset is available in csv format and was generated within the EU CEF VARIoT (Vulnerability and Attack Repository for IoT) project [20]. The IoT traffic-generated sources are listed in [21] and include microcontrollers, smartphones, smart plugs, smart cameras, smart bulbs, smart speakers, smart locks, smoke detectors, motion sensors, access points, and so on. The proposed testbed architecture within the VARIoT project [21] is described below (Figure 4):

### 3.1. IoT Dataset Description

**Figure 5:**
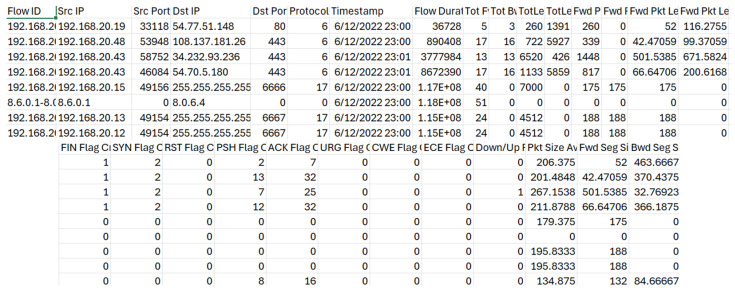
Sample of initial data.

A sample of initially collected data (in the period 6–7 December 2022) is presented in Figure 5. The IoT data collected from different devices was integrated, and the final dataset contained 84 attributes and 35,250 instances.

### 3.2. Data Preprocessing

Data were preprocessed to be in appropriate forms for the learning process. Also, at this stage, data structure was optimized by:

- removing the irrelevant or redundant attributes (*Flow_id* attribute that was composed of other existing attributes),
- adding new attributes that will help the learning algorithms (the *AM_PM* attribute),
- transforming some numerical attributes to nominal ones (*Protocol, PSH_Flags, URG_Flags, FIN_Flags, SYN_Flags*),
- transforming some string attributes to nominal ones (*Src_IP, Dst_IP*).

**Figure 6:**
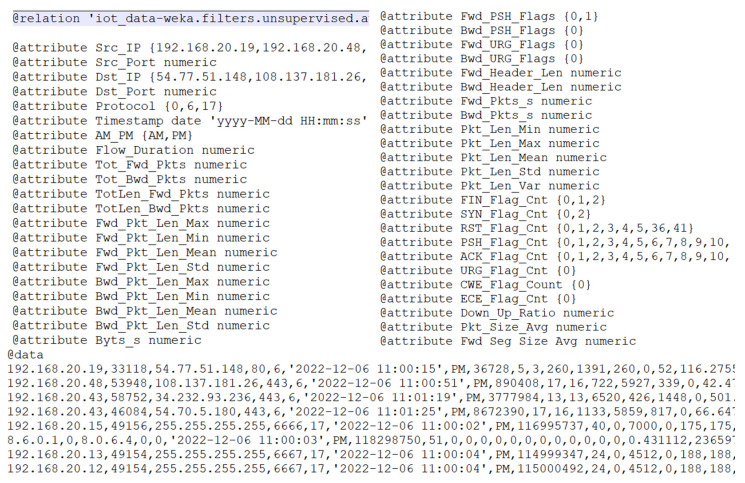
Sample of preprocessed data.

A sample of preprocessed data is presented in Figure 6.

The clustering and classification models learn with high accuracy the attributes that have a known, finite number of values. For this reason, the dataset was analyzed, and such attributes were identified and transformed. 

The values stored within the dataset should be optimum without any redundancy of data. Data redundancy can slow the learning process and can lead to lower rates of accuracy. So, removing the irrelevant and redundant attributes is an important step in the machine learning process. In some cases, by adding new attributes after removing the irrelevant ones, we can help the classifier to better recognize the instances belonging to some weakly represented classes.

For preprocessing and learning data, the Weka Machine Learning Tool 3.9.6 was used [22,23]. The software is open source and contains methods for data preprocessing, data clustering, data classification, data meta-classification, data forecasting, association rules, and data visualization.

### 3.3. Data Clustering

The real-world applications, such as VARIoT data, do not have labels assigned to their instances in order to train classifiers and learn data using intelligent models. For this purpose, a clustering model can be used as a preprocessing stage of the machine learning process. 

After preparing the data for the learning process, partitioning clustering was performed in order to group instances into clusters and to label the instances of the dataset. The k-means algorithm was suitable for this operation, knowing that the number of clusters is equal to 2 (normal traffic anomalies/intrusions traffic) and the groups’ shapes are convex. The Euclidean distance was used for computing the distances between the instances of the dataset. This similarity measure optimally minimized the distances intra-cluster and maximized the distances inter-cluster, discovering well-separated groups of instances. The clustering model was built in 0.13 s (full training data), and the cluster centroids are given below:

Cluster 0 centroid values:

192.168.20.43 46466.0 3.232.21.156 80.0 6 1.670383093E12 AM 9440989.0 2.0 0.0 0.0 0.0 0.0 0.0 0.0 0.0 0.0 0.0 0.0 0.0 0.0 0.211842 9440989.0 0.0 9440989.0 9440989.0 9440989.0 9440989.0 0.0 9440989.0 9440989.0 0.0 0.0 0.0 0.0 0.0 0 0 0 0 64.0 0.0 0.211842 0.0 0.0 0.0 0.0 0.0 0.0 2 0 0 0 2 0 0 0 0.0 0.0 0.0 0.0 0.0 0.0 0.0 0.0 0.0 0.0 1.0 0.0 0.0 0.0 1369.0 0.0 0.0 32.0 0.0 0.0 0.0 0.0 0.0 0.0 0.0 0.0

Cluster 1 centroid values:

192.168.20.42 41631.0 34.104.35.123 443.0 6 1.670378363E12 AM 1.19280365E8 2901.0 20944.0 953.0 2.9520514E7 517.0 0.0 0.328507 10.172748 1420.0 0.0 1409.497422 111.89884 247496.450904 199.907168 5002.531664 82568.080303 2442783.0 13.0 1.19280365E8 41131.160345 234061.655455 2442783.0 30.0 1.19211557E8 5692.191042 88771.996552 2465429.0 40.0 0 0 0 0 92840.0 670216.0 24.320851 175.586317 0.0 1420.0 1238.00499 472.51574 223271.124362 0 2 0 10025 23844 0 0 0 7.0 1238.056909 0.328507 1409.497422 0.0 0.0 0.0 0.0 994.0 1649145.0 0.0 0.0 0.0 1238.0 14600.0 265.0 9.0 32.0 0.0 0.0 0.0 0.0 1.6703856214853298E15 3.5856946416723E7 1.670385682435439E15 1.670385563228757E15

**Figure 7:**
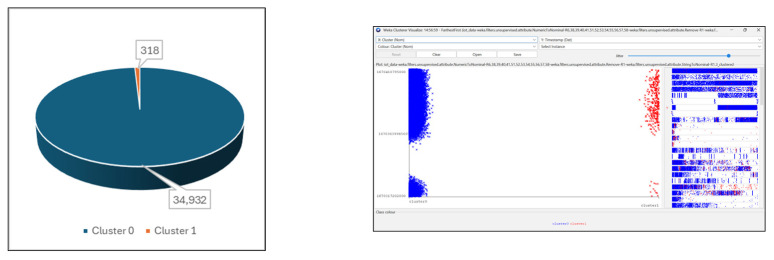
IoT Dataset distribution.

The dataset proved to be unbalanced regarding the distribution of instances into the groups (Table 3 and Figure 7). 

Cluster 0 stores the instances belonging to the normal traffic category (34,932 instances, meaning 99% of dataset instances), while Cluster 1 contains the instances describing the anomalies and intrusions in the network (318 instances, meaning 1% of the total number of instances).

### 3.4. Data Classification

Next, the classification models and performance measures are analyzed from the unbalanced data perspective in order to help the models better recognize the instances of weakly represented classes. These instances are the most important for such applications, being the records with abnormal behavior.

#### 3.4.1. Classification Results for Different Learning Models

**Figure 8:**
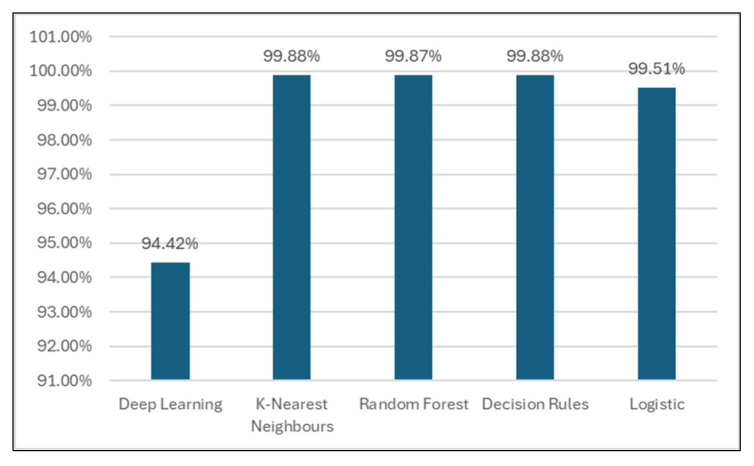
Classification accuracy for different models.

**Figure 9:**
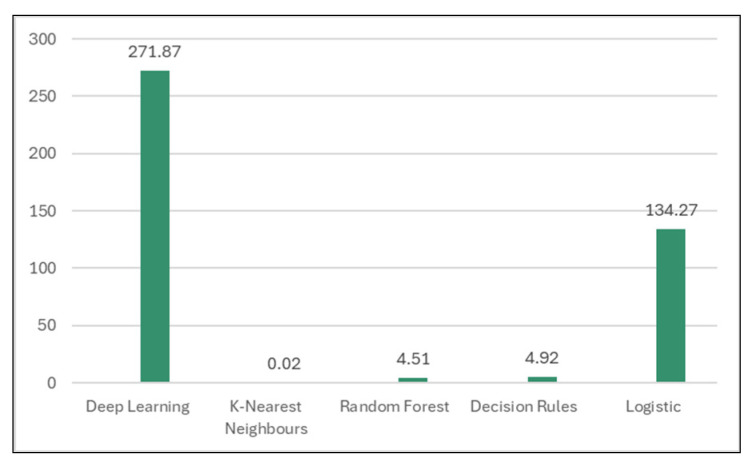
Time taken to build models.

In the classification stage, the dataset was learned by different classifiers (Deep Learning, k-nearest Neighbors, Random Forest, Decision Rules, and Logistics) to decide which is the best model for the considered data. Best accuracy rates (99.88%, Table 4 and Figure 8) were obtained by k-nearest Neighbors and Decision Rules models. K-nearest Neighbors classifier proved an improved time spent to build the model (0.02 s, Table 5 and Figure 9) compared to other models, so this lazy classifier will be used in the next experiments. 

#### 3.4.2. Classification Results for Different K Values (Number of Neighbors)

After choosing the best model, it has to be optimum configured. 

**Figure 10:**
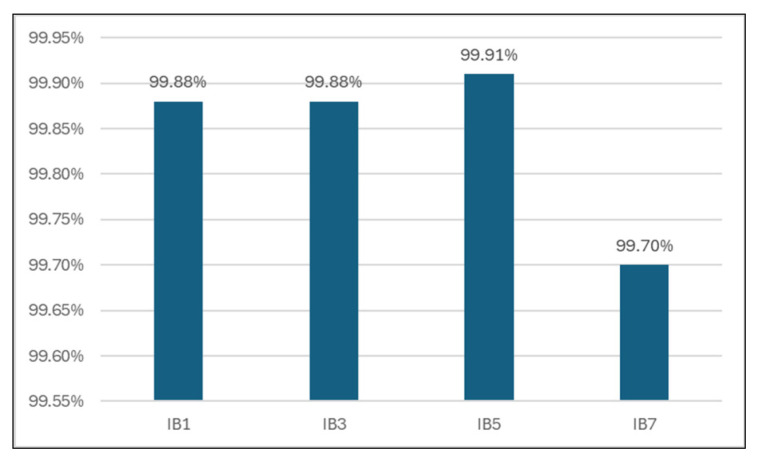
Classification accuracy for different k values.

From Table 6 and Figure 10, we can observe that the classification accuracy reached a peak of maxima for five neighbors (99.91% for k = 5), and then the accuracy records an important drop. 

**Figure 11:**
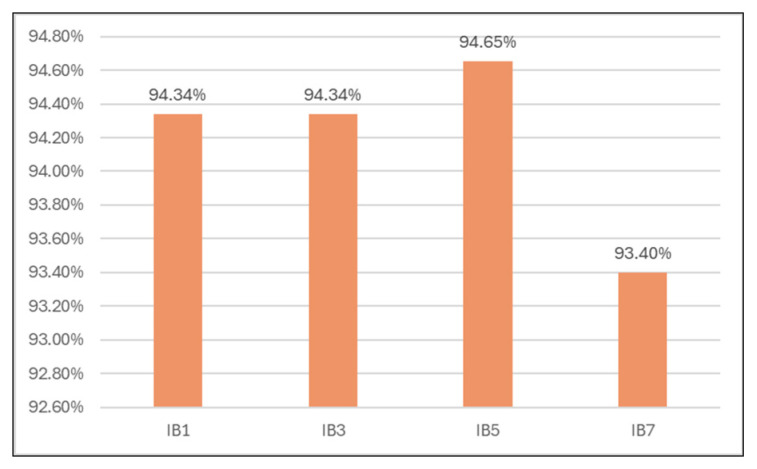
True negative rates for different k values.

Also, the TN rate is maximum for k = 5 (94.65%; see Table 7 and Figure 11). 

**Figure 12:**
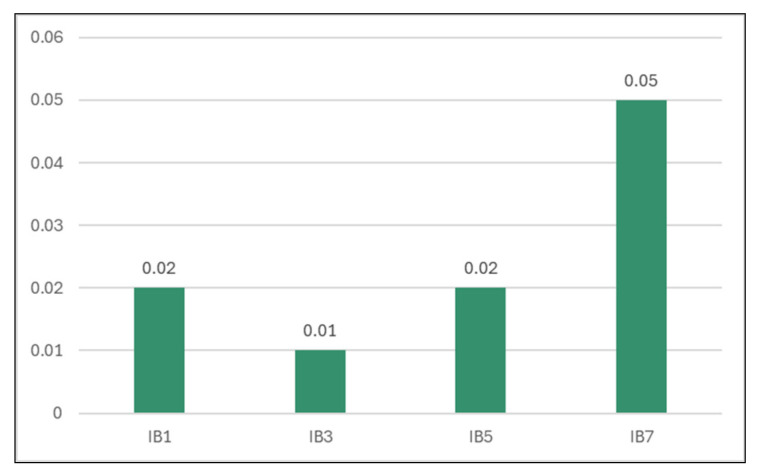
Time taken to build a model for different k values (seconds).

It was also registered as a good time for this model configuration (0.02 s, presented in Table 8 and Figure 12.

#### 3.4.3. Classification Results for Different Distance Functions

The previous models used the Euclidean distance for learning and testing data. 

Also, other distance functions (Chebyshev, Manhattan, Minkowsky) were used to analyze the models’ performance. 

**Figure 13:**
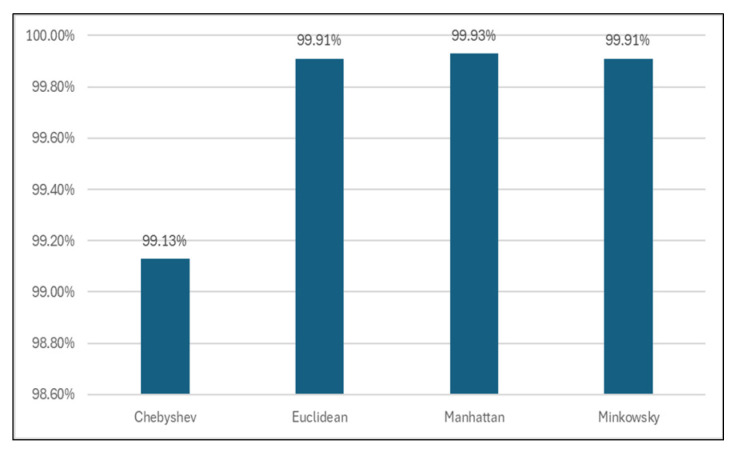
Classification accuracy for different distance functions.

**Figure 14:**
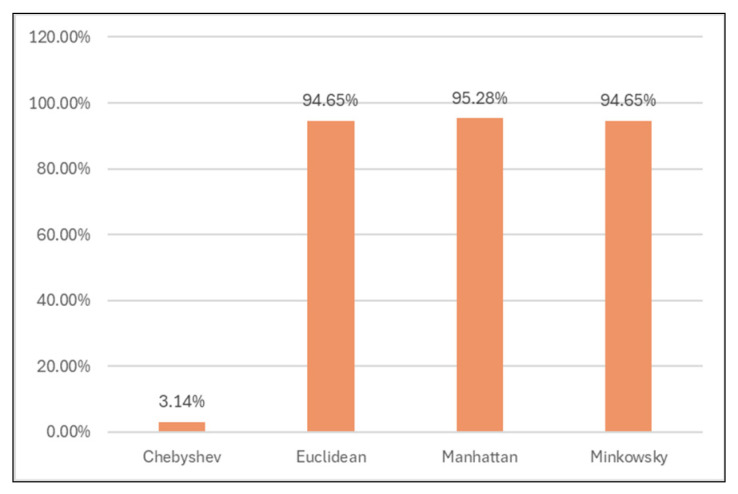
True negative rates for different distance functions.

**Figure 15:**
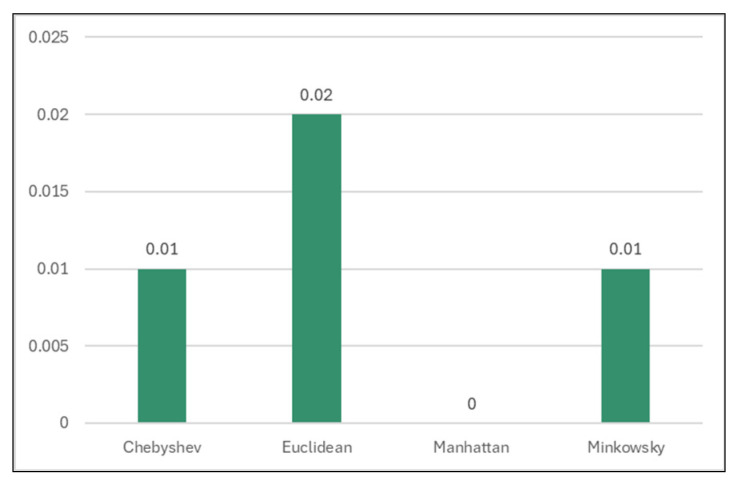
Time taken to build models for different distance functions.

Out of all, Manhattan distance showed improved results regarding classification accuracy (99.93%, Table 9 and Figure 13), TN rate (95.28%, Table 10 and Figure 14), and time spent for generating the models (0 s, Table 11 and Figure 15).

### 3.5. Meta-Classification of Data for Real-Time Intrusions and Anomalies Detection

For unbalanced datasets, the confusion matrix is the most important performance measure because it shows the classification of each class and helps in making decisions that aim for the classification improvement of weakly represented classes. 

In order to analyze the TN rate (meaning the number of correctly classified anomaly and intrusion instances), different cost matrices were used to generate models using a Cost-Sensitive Meta-classifier. As a base classifier, k-nearest Neighbor was applied, with k equal to 5 and the Manhattan distance function. The validation method used in all the performed experiments was 10-fold cross-validation, so all the instances of the dataset were used once in the testing phase and nine times in the training phase, and the average of the ten runs was computed in the end.

**Figure 16:**
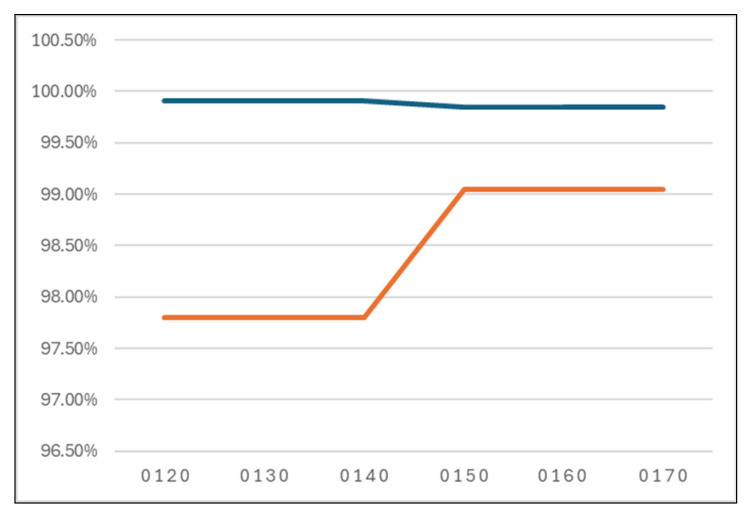
Accuracy and TN rate for different cost matrices.

**Figure 17:**
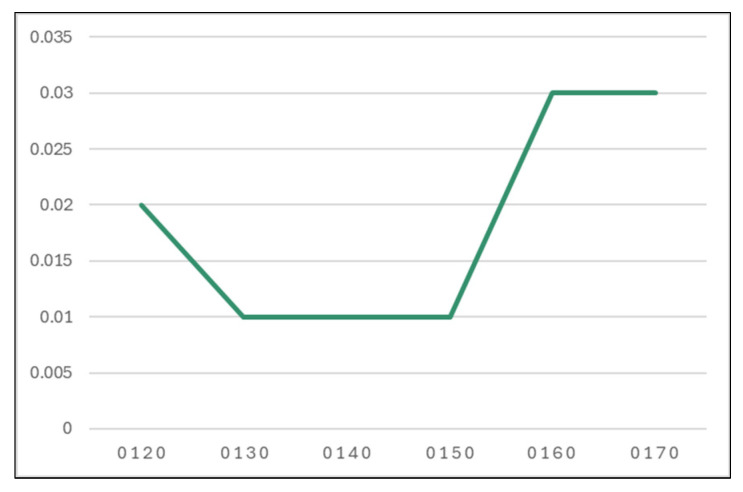
Time taken to build models for different cost matrices.

The experiments showed that improved true negative rates can be obtained by growing the cost of false negative classified instances in the cost matrix. If the maximum TN rate obtained with the base classifier (with a cost matrix equal to 0 1 1 0) was equal to 95.28%, with a cost equal to 2, 3, and 4 for FN instances, a TN rate equal to 97.80% was obtained. Admitting a slow drop in the general accuracy (with a maximum of 0.1% drop), with the cost equal to 5, 6, and 7 for misclassified anomaly and intrusion instances, the TN rate increases to 99.05% (Table 12 and Figure 16). The time taken to build models records the best values (0.01 s) for cost matrices equal to 0 1 3 0, 0 1 4 0, and 0 1 5 0 (Table 13 and Figure 17).

The above results show that the best accuracy rates were obtained with the cost matrix equal to 0 1 1 0, 0 1 2 0, 0 1 3 0, or 0 1 4 0, while the best TN rates were recorded using the cost matrix set to 0 1 5 0, 0 1 6 0, or 0 1 7 0. Also, models were built in optimum time using the cost matrix equal to 0 1 3 0, 0 1 4 0, or 0 1 5 0. To make the best decision in choosing the most suitable model for unbalanced data and real-time learning, an expert system was proposed.

### 3.6. Multi-Agent System for Real-Time IoT Data

For automating the tasks in order to learn real-time data, a multi-agent system is proposed. The proposed agents were developed using the Java Agent Development (JADE) Framework, v. 4.5 [24], integrated with the Weka Machine Learning Tool [22,23].

The Partitioning Clustering Agent’s most important action was to find the best distribution of instances within clusters using the k-means algorithm:
**Partitioning Clustering Agent**1 **Behavior Choose distance function**2     set step = 13     set distance function = "Euclidean"4     set number of clusters = 25     set maximum iterations = 10006     set SSE for Euclidean distance= 0.07     set SSE for Manhattan distance = 0.08 **Set action**9     **switch (step)**10       **case 1:**11       set distance function for K-means equal to Euclidean function12       build K-means model for the given instances13       evaluate K-means model for the given instances14       compute SSE Euclidean for K-means model15       **case 2:**16       set distance function for K-means equal to Manhattan function17       build K-means model for the given instances18       evaluate K-means model for the given instances19       compute SSE Manhattan for K-means model

This agent also starts the classification process by sending the dataset and the “classify” message to Lazy Classification Agent:
**Partitioning Clustering Agent**1 **Behavior Send classify message**2 **Set action**3     send the message “Classify” to Lazy Classification Agent4     send the dataset to Lazy Classification Agent

The Lazy Classification Agent’s main behavior is to classify the data using the optimum parameter values received from its subagents:
**Lazy Classification Agent**1 **Behavior Classification**2 **Set action**3     receive the message “Classify” from Partitioning Clustering Agent4     **if** the message is not null then5       **begin**6       confirm receiving the message7       send reply message ”Classification started”8       train Naïve Bayes classifier9 **end**

The chief agents (Data Preprocessing Agent and Lazy Classification Agent) also have behaviors for communication with their subagents. 

The steps of the learning process can be described as follows:

1. load and visualize the IoT dataset
2. perform the IoT data preprocessing
  - remove the irrelevant attributes of the dataset
  - construct attributes that help the learning process
  - transform some numeric attributes into nominal attributes
  - transform some string attributes into nominal attributes
3. divide the instances into two groups (normal traffic/anomalies and intrusions) using the k-means algorithm
  - search for the best distance function
  - find the best cluster of centroids
4. find the best KNN classification model to predict the class for new instances
  - search for the optimal number of neighbors
  - search for the best distance function
  - evaluate the discovered model using 10-fold cross-validation and the following performance measures: the classification accuracy, time taken to build the model, and true negative rates
5. optimize the classification model
  - learn the Cost-Sensitive metaclassifier
  - find the best cost matrix
  - consult the knowledge base and select the best model by analyzing the performance measures’ values
6. present the best model and the obtained results

By integrating the above steps into a multi-agent system, the IoT data will be learned in real-time using the best-discovered models. The models are optimally configured for unbalanced datasets, being able to identify with high accuracy rates the instances with abnormal behavior.

### 3.7. Expert System for Unbalanced Real-Time Data

For implementing the proposed expert, the CLIPS Building Expert System Tool, v. 6.4.1, was used [25]. 

The designed template contained a slot for each performance measure used for validating the discovered models:

(deftemplate decision 

      (multislot c_m) 

      (slot acc) 

      (slot time) 

(slot tn))

The defined knowledge base consists of facts that describe the results obtained in the meta-classification phase of the machine learning process:

    (deffacts decision_values

       (decision (c_m 0 1 2 0) (acc 99.91) (time 0.02) (tn 97.80))

       (decision (c_m 0 1 3 0) (acc 99.91) (time 0.01) (tn 97.80))

       (decision (c_m 0 1 4 0) (acc 99.91) (time 0.01) (tn 97.80))

       (decision (c_m 0 1 5 0) (acc 99.85) (time 0.01) (tn 99.05))

       (decision (c_m 0 1 6 0) (acc 99.85) (time 0.03) (tn 99.05))

       (decision (c_m 0 1 7 0) (acc 99.85) (time 0.03) (tn 99.05)))

The rules defined in the system knowledge base were fired in order to make some automated decisions regarding the best cost matrix for unbalanced datasets learned in real-time:

      (defrule find-max-acc-tn-time

        (decision (c_m $?c_m1) (tn ?tn1) (time ?time1) (acc ?acc1) )

        (not (decision (tn ?tn2&:(< ?tn2 ?tn1)) (time ?time2&:(< ?time2 ?time1)) (acc ?acc2&:(> ?acc2 ?acc1)) ))

        =>

        (printout t "Cost matrix" ?c_m1 " is the optimum one for tn equal to " ?tn1 ", time taken for building the model equal to " ?time1 " and classification accuracy equal to " ?acc1 crlf))

A sample of results is given below:

The cost matrix (0 1 5 0) is the optimum one for tn equal to 99.05; the time taken for building the model is equal to 0.01, and the classification accuracy is equal to 99.85.

The cost matrix (0 1 4 0) is the optimum one for tn equal to 97.8; the time taken for building the model is equal to 0.01, and the classification accuracy is equal to 99.91.

The cost matrix (0 1 3 0) is the optimum one for tn equal to 97.8; the time taken for building the model is equal to 0.01, and classification accuracy is equal to 99.91.

The cost matrix (0 1 2 0) is the optimum one for tn equal to 97.8; the time taken for building the model is equal to 0.02, and the classification accuracy is equal to 99.91.

The proposed expert system decides what the best parameter values are, finding the best balance between accuracy, time, and classification rates for instances belonging to the underrepresented class. The values that it receives as input are the best performance measures’ values returned by the classification model. 

An important remark is that the system does not allow a drop in the general accuracy greater than 0.1% but finds the optimum cost matrix, taking into account the best classification rate for anomalies and intrusions in the IoT traffic data. Also, the system is designed to work well in real-time detection of abnormal data, given that time is also considered in the proposed expert system. This parameter is important in cybersecurity, being necessary to act quickly when abnormal behavior is detected.

## 4. Discussion

The proposed system is designed to automate all the machine learning tasks for learning IoT data using intelligent agents. One of the most important challenges associated with implementing a multi-agent system for IoT data analysis in smart cities is learning unbalanced datasets and recognizing with high accuracy rates the instances from the underrepresented classes. The proposed architecture is proper for unbalanced datasets, being able to detect anomalies and intrusions in smart city IoT traffic data with high performance. The collected IoT data can be sent to a server where the multi-agent system will be installed; for each new received instance, the system will output, in real-time, the type of instance (normal or anomaly/intrusion). Also, from time to time, the learning models will be updated according to the newly collected data.

## 5. Conclusions

The best machine learning model was discovered using an expert system that finds the best cost matrix for the meta-classification of instances. For detecting with high accuracy, the attacks (meaning high rates for True Negative performance measure, equal to 99.05%), the designed expert admits a small drop in the overall accuracy (0.06%). So, the general accuracy of the best-chosen model is equal to 99.85%, comparable with other results from recent studies for detecting attacks in IoT data. For instance, in paper [4], the authors reported an overall accuracy equal to 99% after learning the data with the Hybrid Convolutional Neural Network model.

As further work, I propose to improve the designed expert system with a fuzzy system in order to automatically discover the thresholds for accuracy and false negative rates allowed drops. In the current research, these thresholds were specified by the user in the expert system by storing them in global constants and by using them in the decision rules of the system. Next, these values can be returned in the defuzzification phase of a fuzzy system after defining fuzzy rules that will be able to describe and learn them.

## Figures and Tables

**Table 1:** Agents’ tasks in the proposed system.

Agent Name	Tasks
Loading Agent	loads the initial dataset containing the IoT traffic generated by the interconnected devices and sensors shows the loaded data sends the dataset to the Data Preprocessing Agent
Data Preprocessing Agent	analyzes and monitors the data preprocessing stage sends the initial and transformed data to Data Preprocessing Subagents receives the preprocessed data from its Subagents sends the final preprocessed dataset to the Partitioning Clustering Agent
Partitioning Clustering Agent	receives the preprocessed dataset builds the optimum k-means model, taking into account different distance functions discovers the optimum cluster distribution (cluster centroids) and labels the instances according to the discovered clusters sends the dataset to the Lazy Classification Agent
Lazy Classification Agent	receives the labeled dataset from the Partitioning Clustering Agent monitors the classification process sends the dataset to its Subagents constructs the optimum k-nearest neighbor classification model, taking into account the optimum number of neighbors and the optimum distance function received from its Subagents sends the dataset and the optimum parameter values for the best-discovered model to the Cost-Sensitive Meta-classification Agent
Cost-Sensitive Meta-classification	receives the labeled dataset and the optimum k-nearest neighbor model configuration from the Lazy Classification Agent performs meta-classification using different cost matrices (considering the confusion matrix generated at each model build) sends the parameters’ values for evaluating the models’ performance, such as classification accuracy, time taken to build the model and true negative rate, together with the cost matrix, to the Expert System Agent receives from Expert System Agent the best cost matrix for identifying with high accuracy rates the anomalies and intrusions in IoT data
Expert System Agent	receives the dataset and the best-discovered models (together with models’ performance in terms of accuracy, time, true negative rate, and cost matrix) from the Cost-Sensitive Meta-classification Agent consults its knowledge base in order to discover the best model for anomalies and intrusions detection in real-time from IoT data send the cost matrix for the optimum discovered model to the Cost-Sensitive Meta-classification Agent
Output Agent	receives the classified dataset and the optimum model configuration from the Cost-Sensitive Meta-classification Agent shows the classification results to the end-user uses the best model in order to discover in real-time the anomalies and intrusions and notifies the end-user when such events appear in the network

**Table 2:** Subagents’ tasks in the proposed system.

Subagent Name	Tasks
Attribute Removal Subagent	identifies the irrelevant or redundant attributes and removes them from the dataset
Attribute Construction Subagent	analyzes if the dataset can be improved by constructing new attributes adds new attributes to the dataset
Numeric_to_Nominal Filtering Subagent	analyzes the domain of values for numeric attributes transforms the numeric attributes into nominal attributes when a small number of values is found in the dataset
String_to_Nominal Filtering Subagent	analyzes the string attributes transforms the string attributes into nominal attributes when a small number of values is found in the dataset
Optimal _no_of_Neighbours Subagent	receives the labeled dataset from the Lazy Classification Agent builds a k-nearest neighbor classification model by varying the value of k (number of neighbors) chooses the best model, taking into account the classification accuracy, true negative rate, and time taken to build the model sends the k value for the chosen model to the Lazy Classification Agent
Optimal_Distance_Function Subagent	receives the labeled dataset from the Lazy Classification Agent builds a k-nearest neighbor classification model by varying the distance function chooses the best model, taking into account the classification accuracy, true negative rate, and time taken to build the model sends the distance function for the chosen model to the Lazy Classification Agent

**Table 3:** Dataset distribution.

Cluster	Number	Percentage
Cluster 0	34,932	99%
Cluster 1	318	1%

**Table 4:** Classification accuracy for different learning models.

Classifier	Accuracy
Deep Learning	94.42%
**K-nearest Neighbors**	**99.88%**
Random Forest	99.87%
**Decision Rules**	**99.88%**
Logistic	99.51%

**Table 5:** Time taken to build classification models.

Classifier	Time
Deep Learning	271.87
**K-nearest Neighbors**	**0.02**
Random Forest	4.51
Decision Rules	4.92
Logistic	134.27

**Table 6:** Classification accuracy for different numbers of neighbors.

Classifier	Accuracy
1-Nearest Neighbor	99.88%
3-Nearest Neighbors	99.88%
**5-Nearest Neighbors**	**99.91%**
7-Nearest Neighbors	99.70%

**Table 7:** True negative rates for different numbers of neighbors.

Classifier	TN rate
1-Nearest Neighbor	94.34%
3-Nearest Neighbors	94.34%
**5-Nearest Neighbors**	**94.65%**
7-Nearest Neighbors	93.40%

**Table 8:** Time taken to build a model for different numbers of neighbors (seconds).

Classifier	Time
1-Nearest Neighbor	0.02
**3-Nearest Neighbors**	**0.01**
5-Nearest Neighbors	0.02
7-Nearest Neighbors	0.05

**Table 9:** Classification accuracy for different distance functions.

Classifier	Accuracy
Chebyshev	99.13%
Euclidean	99.91%
**Manhattan**	**99.93%**
Minkowsky	99.91%

**Table 10:** True negative rates for different distance functions.

Classifier	TN rate
Chebyshev	3.14%
Euclidean	94.65%
**Manhattan**	**95.28%**
Minkowsky	94.65%

**Table 11:** Time taken to build a model for different distance functions.

Classifier	Time
Chebyshev	0.01
Euclidean	0.02
**Manhattan**	**0**
Minkowsky	0.01

**Table 12:** Accuracy and TN rate for different cost matrices.

Cost Matrix	Accuracy	TN Rate
0 1 2 0	**99.91%**	97.80%
0 1 3 0	**99.91%**	97.80%
0 1 4 0	**99.91%**	97.80%
0 1 5 0	99.85%	**99.05%**
0 1 6 0	99.85%	**99.05%**
0 1 7 0	99.85%	**99.05%**

**Table 13:** Time taken to build models for different cost matrices.

Cost Matrix	Time
0 1 2 0	0.02
0 1 3 0	**0.01**
0 1 4 0	**0.01**
0 1 5 0	**0.01**
0 1 6 0	0.03
0 1 7 0	0.03

## Data Availability

The data presented in this study are openly available at https://www.data.gouv.fr/en/datasets/dataset-of-legitimate-iot-data/ (accessed on 27 September 2024) and https://www.data.gouv.fr/en/pages/onboarding/reutilisateurs/ (accessed on 27 September 2024).
